# Effect of inorganic nutrients on bacterial community composition in oil-bearing sandstones from the subsurface strata of an onshore oil reservoir and its potential use in Microbial Enhanced Oil Recovery

**DOI:** 10.1371/journal.pone.0198050

**Published:** 2018-11-29

**Authors:** Thanachai Phetcharat, Pinan Dawkrajai, Thararat Chitov, Pisanu Wongpornchai, Schradh Saenton, Wuttichai Mhuantong, Pattanop Kanokratana, Verawat Champreda, Sakunnee Bovonsombut

**Affiliations:** 1 Interdisciplinary Program in Biotechnology, Graduate School, Chiang Mai University, Chiang Mai, Thailand; 2 Environmental Science Research Centre (ESRC), Faculty of Science, Chiang Mai University, Chiang Mai, Thailand; 3 Northern Petroleum Development Centre (NPDC), Defence Energy Department, Chiang Mai, Thailand; 4 Department of Geological Sciences, Faculty of Science, Chiang Mai University, Chiang Mai, Thailand; 5 Enzyme Technology Laboratory, The National Centre for Genetic Engineering and Biotechnology (BIOTEC), Thailand Science Park, Pathum Thani, Thailand; Argonne National Laboratory, UNITED STATES

## Abstract

Microbial Enhanced Oil Recovery (MEOR) is a promising strategy to improve recovery of residual oil in reservoirs, which can be performed by promoting specific indigenous microorganisms. In this study, we performed preliminary evaluation of the possibility of conducting MEOR at Mae Soon reservoir, an onshore reservoir in Northern Thailand. The reservoir’s physicochemical characteristics, including the characteristics of the wells, the oil-bearing sandstone cores, and the reservoir’s produced water, were determined. The microbiological characteristics of the oil wells in the reservoir were also investigated by submerging the reservoir’s sandstone core samples, obtained from 6 oil wells, in the reservoir’s produced water and in the produced water added with inorganic nutrients (KNO_3_ and NaH_2_PO_4_). The uncultured bacteria in both treatments were determined, using tagged 16S rRNA gene amplicon with Ion Torrent Sequencing Analysis. The effects of inorganic nutrients and the reservoir’s parameters on the bacterial communities were analysed. A total number of 16,828 OTUs were taxonomically classified into 89 classes and 584 genera. In the controls (sandstone cores submerged in the produced water), the dominant bacterial populations were related to *Deinococcus-Thermus*, and *Betaproteobacteria*; while in the nutrient treated samples, there was a marked increase in the relative abundance of *Gammaproteobacteria* in three samples. *Thermus*, *Acinetobacter*, and *Pseudomonas* were the most abundant genera, and these are potential microorganisms for MEOR. Analysis of correlations between physiochemical properties of the reservoir and bacterial genera, using spearman’s correlation analysis, suggested that some of the reservoir’s properties, especially of the well and the rock, could influence some bacterial genera. To our knowledge, this is the first demonstration of the effect of inorganic nutrients on alteration of bacterial communities attached to reservoir’s rock, and how the bacterial, physical, and chemical properties of a reservoir were co-analysed to serve as a basis for designing a MEOR process.

## Introduction

Petroleum resources are becoming more limited, and while other alternative energy resources are being explored, Enhanced Oil Recovery (EOR) technologies are increasingly important. These technologies have been developed in response to the fact that the conventional petroleum recovery procedures can only retrieve 10 to 45 percent of the crude oil [1]. Conventional EOR processes require the use of chemical or thermal methods or miscible gas injection. Some of the factors involved in these processes are harmful to the environment [2]. An EOR method employing microorganisms, known as Microbial Enhanced Oil Recovery (MEOR), offers an alternative way to retrieve residual oil, especially from reservoirs with decreasing productivity, while being environmentally friendly [2]. MEOR has been estimated to increase recovery by up to one-third of the oil initially recovered in an oil reservoir [3]. This technology requires comparatively low amounts of energy to operate and is cost-effective [4].

Many subsurface microorganisms have been reported to play crucial roles in MEOR including *Bacillus*, *Methanobacterium*, and *Clostridium* [5]. Aerobic bacteria, especially *Pseudomonas*, were also found in subsurface strata and often in association with the presence of nitrate. These microbes can function in MEOR through different mechanisms, including the production of gases (CO_2_, CH_4_, H_2_, and N_2_), low molecular weight acids, solvents (especially ethanol and acetone), biosurfactants, and biopolymers. These microbial metabolites can lead to increases in the efficiency of oil recovery by viscosity reduction, permeability alteration, and emulsification of crude oil. The microbial metabolites can decrease the surface and interfacial tensions and alter wettability, resulting in increasing pore scale displacement [3]. One approach for MEOR application is to supply effective microorganism(s) or microbial metabolites from external sources to oil reservoirs [2]. Alternatively, MEOR can employ indigenous microbes, with nutrient supplement as necessary, to increase their metabolic activities, which will enhance oil recovery. For this second approach, microbial communities in the subsurface strata, both the original and the new ones formed after alteration of subsurface conditions, are crucial keys to MEOR success.

Several studies have reported diversity of microbial communities in onshore and offshore oil reservoirs as well as the *in situ* and *in vitro* effects of MEOR on microbial community structures, using culture-dependent methods [6, 7]. However, a large portion of microbes that is indigenous to specific environments such as petroleum reservoirs are non-culturable [5] and, therefore, cannot be detected using the culture-dependent methods. Culture-independent methods provide more comprehensive data for MEOR microbial communities, such as those carried out at the Shengli oil field, China [8], and at the offshore reservoirs in the Norwegian Sea, Norway [9]. Most investigations of microbial communities within a reservoir have been performed using reservoir fluids. There have been very limited studies on microbial communities in oil reservoirs’ rocks. Because most microbes residing in oil reservoirs are attached to the subsurface strata [10], microbial community data obtained from the strata rock samples should be more relevant to MEOR than those obtained from the reservoir fluids or production fluids. Indigenous microbial communities, moreover, could respond to extrinsic nutrient addition in such a way to result in enhanced oil recovery [10, 11]. Inconsistencies have been observed in some previous studies [11] and data on the changes of microbial communities in reservoirs’ rock resulting from nutrient addition are still lacking.

With these gaps in MEOR studies, we therefore aimed to conduct an investigation on the diversity of bacteria derived from the oil-bearing rock regions of a reservoir using Ion Torrent Sequencing Analysis, to demonstrate how an *in vitro* nutrient treatment could affect bacterial community structures, and to analyse the correlations between some key characteristics of the reservoir and its indigenous bacterial communities. These investigations were not only intended to provide a basis for future *in situ* MEOR implementation at the study site (Mae Soon Reservoir, Fang Basin, Thailand), but also to demonstrate how the bacterial structure and the additional inorganic nutrients together with the physicochemical characteristics of a reservoir, are related and how these parameters can be co-analysed and manipulated, in order to serve as a basis for designing a MEOR process.

## Materials and methods

### Ethics statement

All samples were collected within territories governed by the NPDC, Defence Energy Department (Mae Soon reservoir of Fang oil field, Chiang Mai, Thailand), with the permission granted through Capt. Pinan Dawkrajai, RTN, Ph.D., (Director of Exploration and Production Division, NPDC, Defence Energy Department).

### Core sample collection

**Fig 1:**
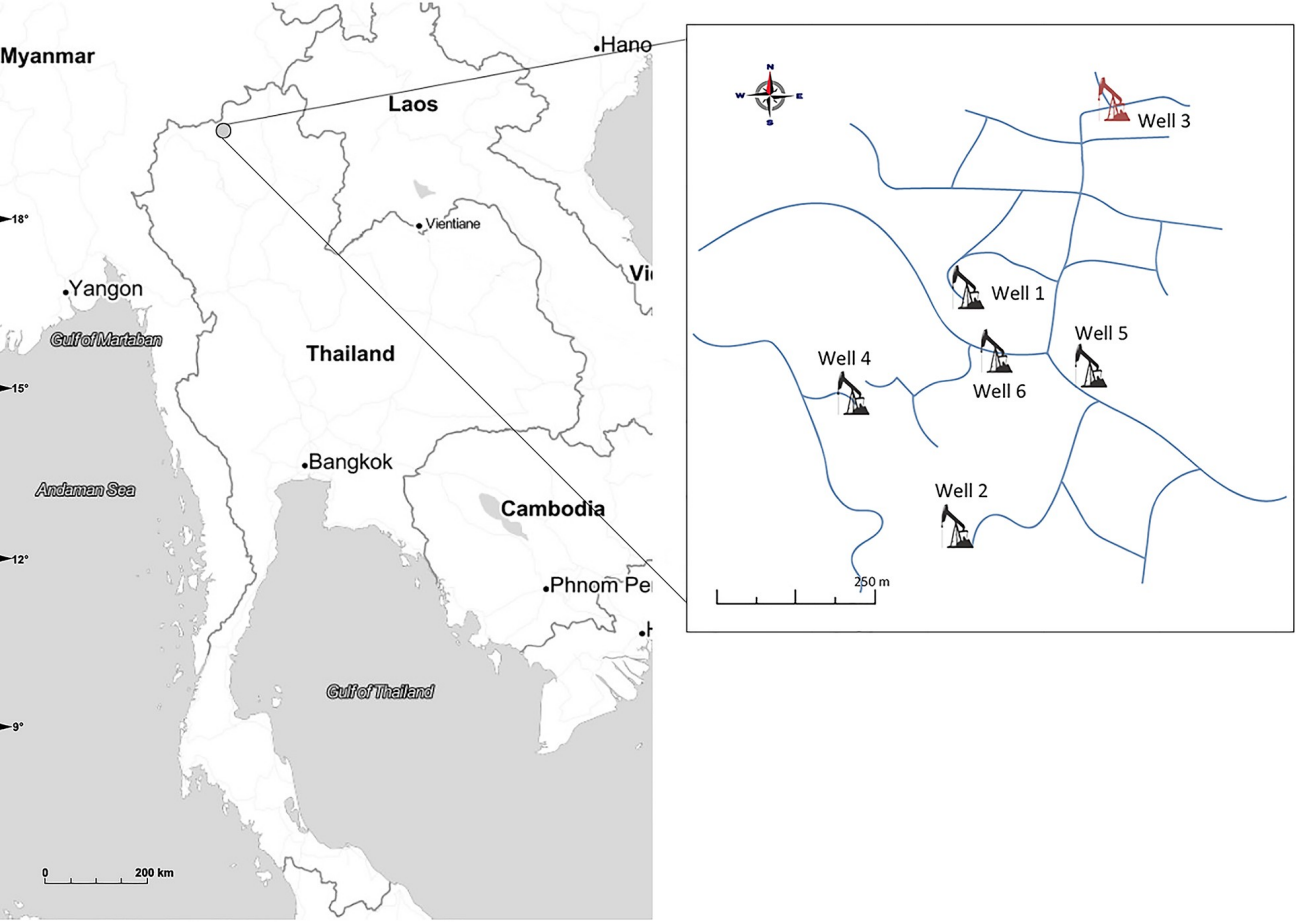
Location and distribution of six oil wells in Mae Soon reservoir, Fang oil field, Thailand.

Core samples used in this study were obtained from the reference core collection of Mae Soon reservoir of Fang oil field, Chiang Mai, Thailand (19°50'N, 99°09'E) (Fig 1). This reservoir has been in production for more than 50 years, with average productivity of 900 barrels of oil per day (bbl/d), and is currently experiencing a decline in its production rate. Six oil-bearing sandstone samples were aseptically drawn from the six-foot-long cylindrical cores drilled from 6 oil wells in the reservoir. These oil-sand samples were from the depths of 609–756 metres below the earth’s surface.

### Reservoir characterisation

Temperatures and depths of oil reservoirs were retrieved from the well logs. Porosity, permeability and grain density of the core samples were determined according to the routine core analysis (performed by GENLabs, Thailand). The lithology of the core rock was analysed using an X-Ray fluorescence (XRF) spectrometer [12].

Produced water (the water found in the same formations as oil and gas, which is brought to the surface with the petroleum products) from the production wells was analysed for pH and salinity using a standard pH meter (Ohaus starter 3100, USA) and an electrochemical analyser (Consort C933, Belgium). Concentrations of sulfate and nitrate in the produced water were determined using ion chromatography (Dionex ICS-3000, USA). Iron content was analysed using inductively coupled plasma optical emission spectrometry (ICP-OES) [13].

### Inorganic nutrient enrichment

To study the effect of inorganic nutrients on the changes in microbial composition within the oil sand samples, each of the six oil-bearing sandstone samples was extracted from the drilled core. Only the inner part of each core was sampled; it was placed in a sterile plastic bag, then crushed using a hammer, mixed, and divided into two 10-gram portions. The first portion was placed in a 250-ml screwed-cap DURAN bottle which was then filled completely with the produced water (collected in sterilised containers from the pipeline connecting to the reservoir, heated to 60°C prior to use). The cap was then tightened to create a hypoxic condition (with DO of 0.8 mg/L, measured using a HI 98194 Multiparameter Waterproof Meter, Hanna instruments, Romania). This treatment was performed to resemble the conditions in the formation rock layer where the core originated. The samples treated with the produced water (assigned C1P –C6P) were used as control samples. Another portion of each oil sand sample was flooded with the produced water supplemented with inorganic nutrients (0.12% (w/v) KNO_3_ and 0.034% (w/v) NaH_2_PO_4_, prepared from AR grade chemicals (RCI, Labscan, Thailand)), based on a core flooding protocol previously reported to be effective for oil recovery [14]. The nutrient was added to the produced water just prior to conducting the experiment. These were assigned nutrient-treated samples (C1N –C6N); they were treated under the same elevated temperature and hypoxic condition as the controls). The controls and the nutrient-treated samples were incubated at 60°C in a water bath (Julabo, Germany) for 32 weeks.

### DNA isolation and tagged 16S rDNA gene sequencing

The enriched core samples were separated from the fluids by centrifugation at 4,766 g for 5 min. The genomic DNA was extracted from 10 grams of the samples using 10 ml of lysis buffer (100 mM Tris-HCl, 100 mM Na-EDTA, 100 mM NaPO_4_, pH 8.0, 1.5 M NaCl and 1% (v/v) CTAB) and 100 μl of proteinase K [15]. The solution was shaken horizontally at 37°C for 40 min. After that, 1 ml of 20% (w/v) SDS was added, and the solution was further incubated at 65°C for 30 min. The mixture was then centrifuged at 4,766 g for 5 min at room temperature. The aqueous phase was collected and extracted with chloroform/isoamyl alcohol 24:1 (v/v) and centrifuged at 4,766 g for 10 min. Genomic DNA was precipitated with 0.6 volume of isopropanol at room temperature for 15 min, and the pellet was collected after centrifugation at 13,088 g for 20 min. The DNA pellet was washed with cold 70% (v/v) ethanol and dried at room temperature.

The purified metagenomic DNA was used as a template for amplification of the partial 16S rRNA gene. Universal bacterial primers E785F (5′-GGATTAGATACCCTGGTAGTCC-3′) and E1081R (5′-CTCACGRCACGAGCTGACG-3′) attached with tagged barcode sequences [16] were used for amplifying the V5 and V6 regions of prokaryotic 16S rRNA genes. Polymerase chain reactions were performed using Phusion High-Fidelity DNA polymerase (Thermo Scientific, Massachusetts, USA) on a MyCycler thermocycler (Bio-Rad, Hercules, CA) under the following conditions: initial denaturation at 98°C for 30 sec, 25 cycles of denaturation at 98°C for 10 sec, annealing at 66°C for 30 sec, and extension at 72°C for 30 sec, with a final extension at 72°C for 5 min. The PCR products were analysed on a 2% (w/v) agarose gel and purified using the GF-1 ambiclean kit (Vivantis, Malaysia). Then, all amplified products were quantified using a Nanodrop spectrophotometer (LabX, CA). The sequences were analysed using the ION PGM system with ion 316 chip kit V2 (Life Technologies, CA, USA) following the manufacturer’s recommended protocols. All 16S rRNA gene sequences of the oil sand samples were deposited in the NCBI Sequence Read Archive (SRA) with the project accession number SRP071710.

### Data analysis

The sequencing dataset was initially processed by removing low-quality score reads with a cutoff of 20 for the Phred quality score. The sequences were demultiplexed into specific groups based on the barcode sequences and then the tagged and primer sequences were trimmed off using QIIME (Quantitative Insights into Microbial Ecology) version 1.9.1 [17]. The sequences were clustered into Operational Taxonomic Units (OTUs) for classified taxon and alpha diversity analysis. Chimeric sequences were detected and removed by UCHIME [18], based on the referenced dataset from the Ribosomal Database Project [19]. The remaining sequences were clustered through an open-reference method using UCLUST at 97% similarity [20], with the minimum OTU size set to 2 sequences in order to exclude singletons. The representative sequence of each cluster was determined taxonomically based on the nomenclature from the Greengene database [21]. To compare the bacterial diversities among the samples, alpha diversity index including observed OTUs, Shannon-Weaver index, and the Chao1 richness estimator were calculated from the OTU Table. Principal coordinate analysis (PCoA) was used to illustrate the beta diversity of bacterial communities using the unweighted Unifrac matrix [22]. The differences in bacterial profiles were analysed using statistical analysis of taxonomic and functional profiles software (STAMP) [23]. **The** correlations between some physical and chemical factors of the reservoir and microbial genera were analysed using Spearman’s Correlation [24].

## Results

### Physical characteristics of the reservoir

A previous survey has shown that the formation pressure of the deepest oil-bearing zone of this reservoir was 1,082 psi (unpublished data, provided by Northern Petroleum Development Centre). The physical characteristics of the sandstone cores, together with the physical properties of the oil wells from which the cores originated are summarised in Table 1. It can be seen that the lithological nature of the cores, their porosity and grain density were slightly different, but the degrees of permeability, which reflect the mobility of petroleum liquid, had the greatest variation among the samples.

**Table 1:** Characteristics of core samples and the wells from which core samples originated.

sample	core characteristics				well characteristics	
	description	porosity (%)	permea- bility (mD)	grain density (g/cm^3^)	formation temper-ature (°C)	depth (metres)
C1	Conglomerate, dark gray, consolidated, fine-grain matrix, non-calc	11.20	0.10	2.64	71	741
C2	Sandstone, dark gray, medium-coarse grained, well sorted, white argillaceous matrix, consolidated, non-calc, trace coal, trace black lithic fragments	23.00	193.00	2.63	68	666
C3	Sandstone, dark gray, fine grained, well sorted, argillaceous matrix, consolidated, non-calc, interbedded with clay	20.80	110.00	2.64	72	756
C4	Sandstone, dark gray, medium-coarse grained, poorly sorted, clay matrix support, consolidated, non-calc, trace coal, trace micro fracture	21.10	47.60	2.63	63	609
C5	Sandstone, dark gray, very coarse grained, poorly sorted, clay matrix support, consolidated, non-calc, trace coal, lithic fragments	13.40	0.29	2.63	68	659
C6	Sandstone, dark gray, medium-coarse grained, poorly sorted, clay matrix support, consolidated, non-calc, trace coal, trace micro fracture	nd	nd	nd	67	644

nd: not determined since complete core plug could not be accessed for these analyses.

### Chemical characteristics of the oil-bearing sandstone cores and produced water

The chemical properties of the oil-bearing sandstone cores and the produced water were analysed in order to evaluate the possibility of applying MEOR to the oil wells in this reservoir. Elemental compound analysis, using an X-ray fluorescence spectrometer, revealed that the cores from this sandstone-based reservoir consisted mainly of SiO_2_ (80–85%). Elements including nickel, vanadium, rubidium, yttrium, niobium, chromium, strontium, barium, and zirconium were present in all samples. The chemical properties of the cores are summarised in Tables 2 and 3.

**Table 2:** Percentages of elemental compounds of the core samples.

core sample	compound proportion (%)										
	Al_2_O_3_	Fe_2_O_3_	K_2_O	MgO	MnO	Na_2_O	P_2_O_5_	SiO_2_	TiO_2_	CaO	Loss on ignition+SO_3_
C1	6.64	1.92	1.81	0.37	0.03	0.13	0.06	86.09	0.40	0.10	2.67
C2	6.09	1.33	2.79	0.19	0.03	0.14	0.06	85.19	0.21	0.08	4.18
C3	10.29	2.36	2.04	0.72	0.04	0.24	0.08	78.49	0.55	0.10	5.09
C4	5.64	2.48	1.70	0.31	0.07	0.16	0.09	82.54	0.37	0.16	6.63
C5	11.43	2.56	2.40	0.85	0.03	0.14	0.11	77.00	0.61	0.12	4.81
C6	5.03	1.04	2.22	0.15	0.03	0.13	0.04	88.42	0.16	0.05	2.77

**Table 3:** The element contents of core samples.

core sample	element content (ppm)								
	Ni	V	Rb	Y	Nb	Cr	Sr	Ba	Zr
C1	47.89	70.54	97.00	5.04	bd	136.35	31.07	155.43	194.60
C2	36.10	44.87	147.75	2.99	bd	205.60	44.47	295.04	116.06
C3	48.84	95.02	121.50	7.22	0.11	125.00	23.45	100.90	191.30
C4	41.16	68.93	95.59	4.50	bd	165.14	23.32	103.55	214.38
C5	53.23	110.18	133.03	7.96	1.89	143.38	41.08	141.40	238.69
C6	30.16	32.20	117.61	4.58	bd	146.54	36.00	257.88	106.56

bd: below detection limit.

Produced water from the oil wells was analysed for chemical properties, including pH and salinity. In addition, the concentrations of sulfate, nitrate, and iron, which can be the sources of nutrient for microorganisms, were also determined. We found that the produced water from most wells (excluding well 3, which is presently abandoned) does not vary significantly in salinity, which is in the normal range of the salinity of onshore reservoirs. The pH of the produced water from the 6 wells tested showed that the wells were slightly alkaline. Sulfate was not detected in the produced water from most wells, while nitrate was present in every well, and iron in 4 out of 5 wells (Table 4). An analysis of a representative crude oil sample from Mae Soon oil field showed that it had an API gravity of 30°, a viscosity of 22 Cp (at 50°C), and 18 (wt%) wax (unpublished data, provided by Northern Petroleum Development Centre).

**Table 4:** The salinity, pH and the dissolved compound concentrations of produced water.

sample	salinity (ppm)	pH	SO_4_ (mg/L)	NO_3_ (mg/L)	Fe (mg/L)
PW1	600	7.8	bd	0.76	0.10
PW2	300	7.2	4.99	0.31	1.85
PW4	500	8.6	bd	0.40	bd
PW5	600	8.0	bd	0.34	0.46
PW6	300	7.5	bd	0.24	1.94

bd: below detection limit.

### Characterisation of microbial diversity of the core samples

**Fig 2:**
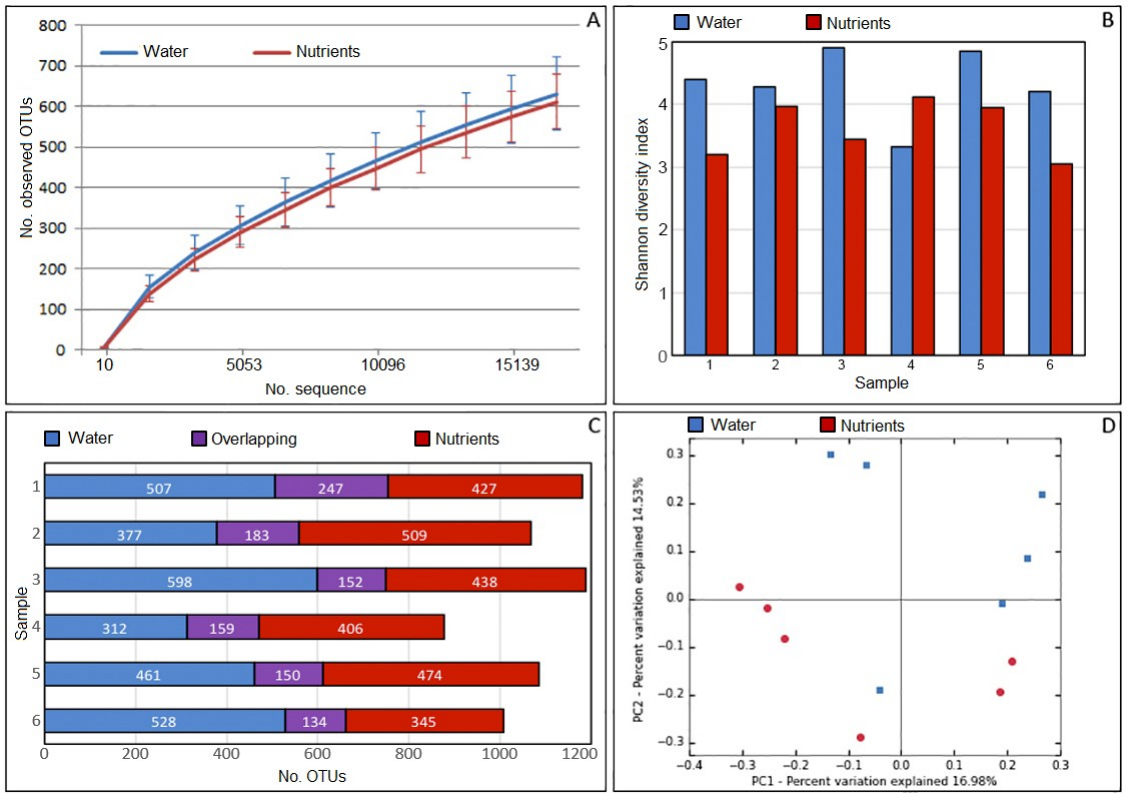
The dataset and diversity indices of metagenomic sequences. (A) Rarefaction curves of observed OTUs for the control and nutrient-treated groups, (B) Shannon diversity index comparison between the two treatments, (C) The number of unique and shared OTUs between the two treatments, (D) The principal coordinate analysis (PCoA) plot showing differences in bacterial community between the control and the nutrient-treated groups.

The purified DNAs from the sandstone core samples treated with produced water (C1P-C6P; control samples) and from those treated with produced water supplemented with inorganic nutrient (C1N-C6N) were sequenced. A total of 1,426,176 filtered reads were obtained from the ION PGM sequencing. The number of reads obtained from each sample ranged from 17,039 to 220,923 reads. The Operational Taxonomic Units (OTUs) in the samples were assigned from the filtered sequences at 97% similarity levels. The bacterial phylotypes ranged from 471 to 754 OTUs at the genetic distance of 0.05 with an average of 619 OTUs per sample. Rarefaction analysis was used to determine the microbial diversity in the filtered data set. The curves show similar numbers of OTUs obtained from both treatments, indicating that there was no significant effect of treatment on the numbers of OTUs as assessed by rarefaction (Fig 2A and S1 File). In order to reduce the bias of unequal number of sequences, normalisation was performed by subsampling method at 16,828 sequences per sample. The highest bacterial richness was observed in sample C1P while the lowest one was found in C4P. Meanwhile, Chao1 estimator indicated that samples C5N and C6N contained the highest and the lowest numbers of OTUs, respectively. According to Shannon’s diversity indices, greater bacterial diversities were observed in the controls than in the nutrient-treated samples, with the exception of sample C4, in which bacterial diversity increased after being treated with inorganic nutrient (Mann-Whitney U test; p-value = 0.026, see in S1 File) (Fig 2B). The diversity indices are summarised in Table 5.

**Table 5:** Numbers of sequences, OTUs, and alpha diversity indices of the bacterial community in the sand core samples treated with oil well produced water and produced water supplemented with inorganic nutrients.

treatment	sample	no. of raw sequence	observed OTUs*	chao 1 Index*
samples submerged in produced water (controls)	C1P	86,125	754	1,621
	C2P	204,309	560	1,331
	C3P	103,723	750	1,542
	C4P	194,305	471	1,194
	C5P	17,039	611	1,125
	C6P	58,578	662	1,465
samples submerged in produced water supplemented with inorganic nutrients (nutrient-treated samples)	C1N	70,421	674	1,361
	C2N	55,916	692	1,321
	C3N	192,276	590	1,375
	C4N	125,232	565	1,356
	C5N	220,923	624	1,624
	C6N	97,329	479	988

*calculated from rarefied OTUs at 16,828 sequences per sample.

According to the shared OTUs chart (Fig 2C), bacterial groups in the sandstone core were altered after being treated with inorganic nutrients. Smaller numbers of shared OTUs between the two treatments were observed compared to unique OTUs found in either produced water-treated or nutrient-treated samples. More than one thousand unique OTUs were found in produced water-treated samples C1P –C4P and nutrient-treated samples C3N –C5N. Principal coordinate analysis (PCoA) was performed to review the relationship of bacterial communities in the cores subjected to the two treatments (S1 Table). PCoA indicated that the bacterial community profiles were associated with the treatment conditions used (Fig 2D).

### Bacterial community structures in oil-bearing sandstone cores treated with produced water and with additional inorganic nutrients

**Fig 3:**
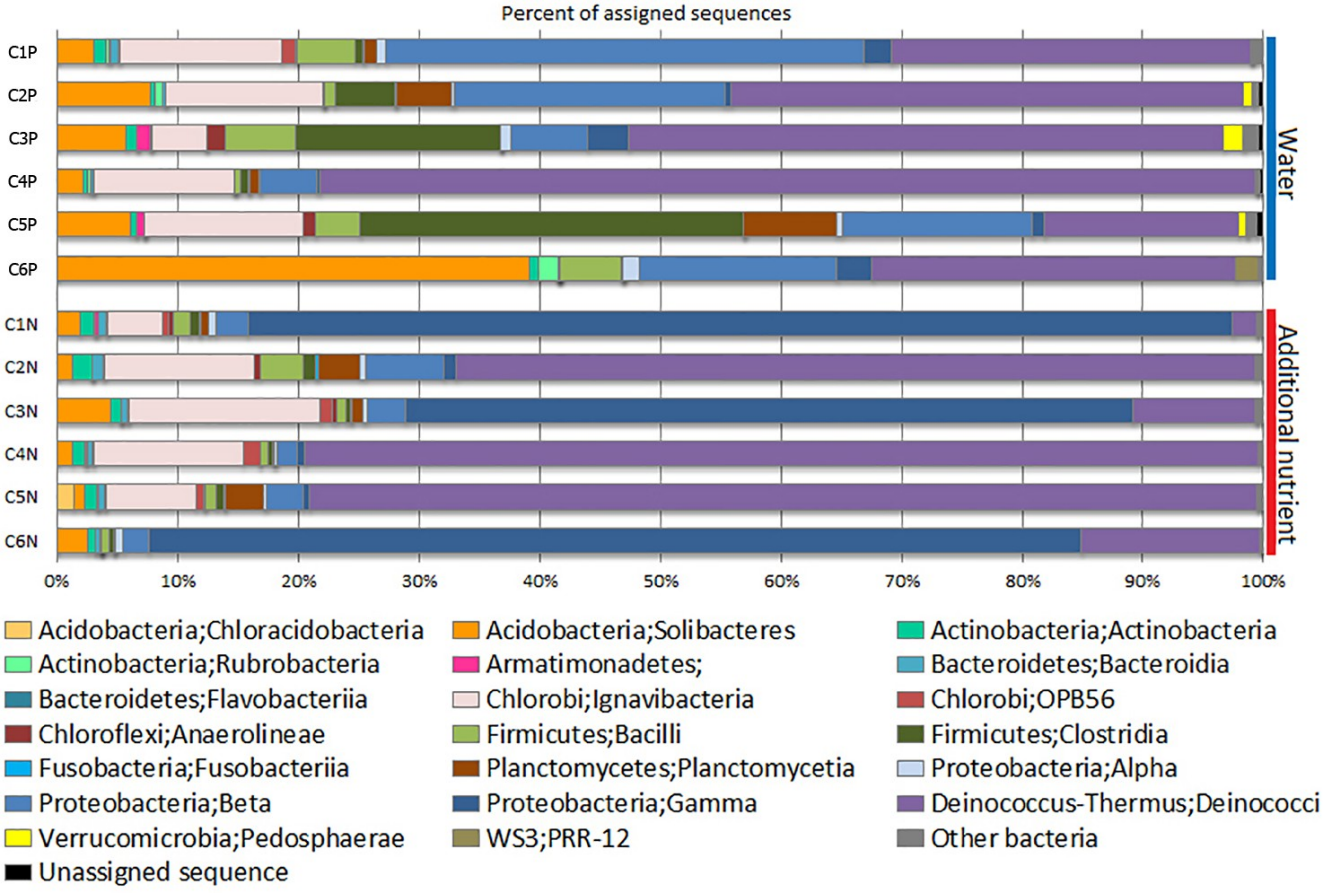
Class-level classification of bacteria in the control and the nutrient-treated sandstone core samples.

Bacterial community profiles of the two treatments were analysed at the class level (Fig 3). From Fig 3, diverse bacterial groups were observed in both treatments. In the controls, bacteria in classes *Deinococcus–Thermus*, *Proteobacteria* (Beta), *Acidobacteria* (Solibacteres), and *Firmicutes* (Clostridia) were the predominant classes having mean proportions of 40.9%, 17.5%, 10.7%, and 9.2%, respectively. When the samples were treated with inorganic nutrients, the predominant classes were mainly *Deinococcus–Thermus* and *Proteobacteria* (Gamma). *Deinococcus–Thermus* was the major taxon found with the proportion of 41.8%, while *Proteobacteria* (Gamma) was the second most abundant class, contributing 36.9%. *Acidobacteria*, *Proteobacteria* (Beta), and *Firmicutes* (Clostridia) were found only in small proportions in the nutrient-treated samples. If considered by means of individual samples, there was a significantly increase (P < 0.05) in the relative abundance of *Gammaproteobacteria* in three samples (C1N, C3N, and C6N) obtained from three individual wells (well 1, 3, and 6), as a result of the inorganic nutrient treatment.

At the order level, the top five orders in the control group, according to the OTU abundances, were *Thermales* (in C4P, C3P, and C2P), *Solibacterales* (in C6P), *Burkholderiales* (in C1P and C2P), *Clostridiales* (in C5P), and SM1H02 (in C1P, C5P, and C2P). For the nutrient-treated group, the top five orders were *Pseudomonadales* (in C1N, C6N and C3N), *Thermales* (in C4N, C5N, and C2N), SM1H02 (in C3N, C2N, and C4N), *Solibacterales* (in C3N), and *Pirellulales* (in C2N and C5N), although the latter two had abundances smaller than 10%.

**Fig 4:**
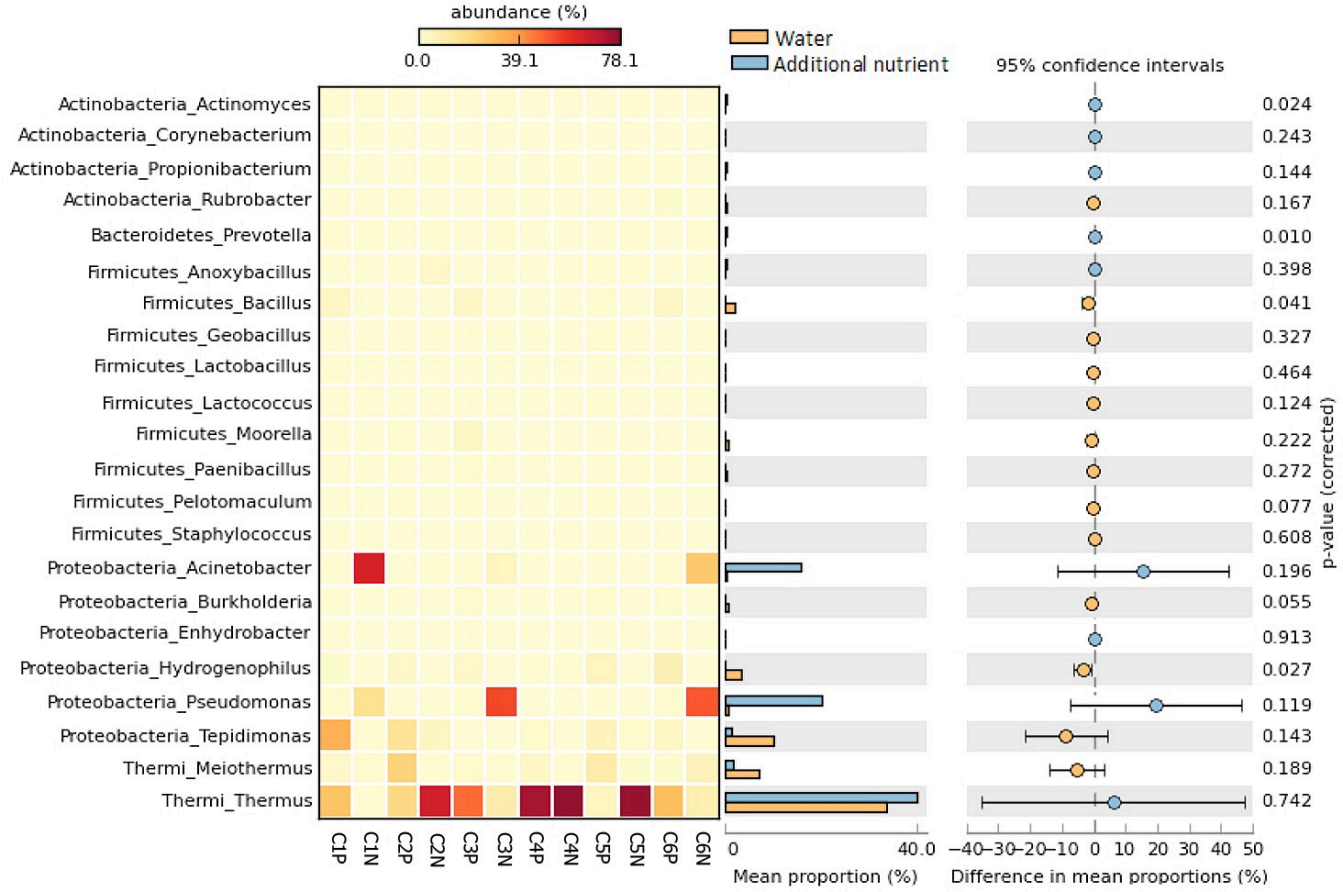
Comparative analysis of genus abundance in bacterial communities from the controls (C1P-C6P) and nutrient-treated samples (C1N-C6N).

We also determined the effects of different treatment conditions on bacterial diversity at the genus level. The abundances of bacterial genera were different between the samples submerged in produced water and those treated with inorganic nutrients (Fig 4). In the overall picture of the reservoir, *Thermus* was the most abundant genus, both in the samples submerged in produced water and in the samples treated with additional nutrients, having average distributions of 33.8% and 40.0%, respectively. Treatment with the inorganic nutrient caused a slight increase of this genus (6.2%). The populations of this genus increased in samples C2N (20.0% to 65.9%), C4N (74.5 to 78.1%), and C5N (5.2% to 76.8%); but decreased in samples C1N (27.3% to 0.6%), C3N (47.3% to 9.9%), and C6N (28.5% to 8.6%). Other bacterial genera that were obviously altered as a result of nutrient treatment were *Pseudomonas* and *Acinetobacter*. *Pseudomonas* increased up to 19.7%, while *Acinetobacter* increased up to 15.5%. Alterations of these genera were not consistent in all samples, although they increased in the majority of the samples. For example, the relative abundance of *Pseudomonas* increased in nutrient-treated samples C1N, C3N, C4N, and C6N. *Acinetobacter* increased in C1N, C2N, C3N, C4N, and C6N. The most prominent increase in *Acinetobacter* was observed in nutrient-treated sample C1N (from 0.6% in C1P to 63.9% in C1N; an increase of 63.3%), and *Pseudomonas* in C3N (from 0.9% to 53.8%; an increase of 52.8%).

On the other hand, some genera, such as *Tepidimonas*, *Meiotermus*, *Hydrogenophilus*, and *Bacillus*, experienced average decreases in their proportions after nutrient treatment (Fig 4). The relative abundance of *Tepidimonas* decreased most obviously in the sample C1 (from 32.9% (C1P) to 0.6% (C1N)), *Meiothermus* in C2 (from 22.5% (C2P) to 0.4% (C2N)), *Hydrogenophilus* in C6 (from 7.9% (C6P) to 0.001% (C6N)), and *Bacillus* in C6 (from 3.7% (C6P) to 0.08% (C6N)).

### Correlations between physicochemical properties and bacterial genera

Spearman’s correlation analysis showed statistically significant correlations between physicochemical factors and bacterial genera in the reservoir communities (P-value < 0.05) (see S2 Table for details).

Concerning the well characteristics, the formation temperature and well depth had effects on the proportions of some bacterial genera, especially *Corynebacterium* in the controls and *Lactobacillus* in the nutrient-treated samples. The higher the temperatures and the larger the well depths, the more abundant these genera were.

As for the core characteristics, several factors, including physical and chemical characteristics, were found to affect bacterial genera in the communities. Among the physical factors, grain density positively correlated with the proportions of *Acinetobacter*, *Bacillus*, *Corynebacterium*, *Enhydobacter*, and *Streptococcus* (in the controls; C) and *Lactobacillus* (in the nutrient-treated samples; N); while permeability had a negative correlation with *Rubrobacter* (N). Porosity, however, did not have significant correlation with any bacterial genera. Among the chemical compositions of the reservoir rocks, there were connections between some elements and compounds such as Cr, MnO, P_2_O_5_, and Na_2_O and some bacterial genera, which were mostly in negative correlation patterns. Cr and MnO had effects on *Acinetobacter*, *Corynebacterium*, and *Enhydobacter* (C), P_2_O_5_ on *Enhydobacter* and *Acinetobacter*, and Na_2_O on *Tepidomonas* and *Lactobacillus* (C) and *Bacillus* (N). Some positive correlations were observed, such as those between P_2_O_5_ and *Thermus* (N), but such correlations were exceptions.

The produced water, in contrast to the well and rock properties, had no significant correlation with bacterial genera for both treatments.

## Discussions

Mae Soon reservoir is one oil-bearing reservoirs in the Fang oil field, with the oil sand at a depth between 609 and 756 metres. The depth affects the temperature and pressure of the formation. The temperature, in particular, can affect microbial growth and metabolism [5]. Because Mae Soon reservoir is a shallow reservoir, the formation temperature is not too high (63 to 72°C) and this range of temperature can support growth and metabolism of moderate thermophiles [25]. The formation pressure of the deepest oil-bearing zone of Mae Soon is 1,082 psi, which should not limit bacterial metabolism, according to Donaldson *et al*. (1989), who report that pressure lower than 1,400–2,800 psi does not affect on bacterial metabolism [5].

The physical and chemical properties of the oil-bearing rock and the properties of the produced water from oil wells within a reservoir can determine the success of the MEOR method. Lithological analysis revealed that the rock of reservoir was sandstone-based. According to Brown *et al*. (2000), sandstone-based rock is favourable for the biotechnological oil-recovery method [10]. Core analysis showed that different sandstone core samples varied greatly in their degrees of permeability, which ranged from less than 1 to over 100 mD. Permeability of less than 75 mD is expected to limit effective microbial transport through the reservoir [26]. Thus, from the MEOR perspective, the permeabilities of core samples C2 and C3 seemed to be promising. As for the porosity, which reflects the fluids (water or hydrocarbons) contained within the reservoir rock [26], the values were within the range found in most commercial petroleum reservoirs, ranging from 10–25%. This parameter might affect the cell transport in the pore space of the oil reservoir [5]. Iglauer *et al*. (2010) observed that rocks with porosity of about 20% supported recovery of additional incremental oil from the sandstone core up to 75% [27]. The cores contained elements commonly found in other sandstone reservoirs’ strata [28]. Moreover, the produced water, which was low in salinity and had neutral to slightly alkaline pH, could support microbial growth [5]. The crude oil (which in Mae Soon reservoir includes paraffin) could be used by microorganisms as a carbon source. The oil API gravity and its viscosity are also within the acceptable ranges for MEOR processes [29, 30].

Besides the characteristics of the well, the rock, and the produced water, the microbiological characteristics of the reservoir were investigated. The experiment of enrichment process of 8 month period was designed as a laboratory trial to observe what might happen to the bacterial communities residing the well/reservoir rock after being treated with inorganic nutrients for an extended period of time. This enrichment process was designed according to a field experiment proposal, using enrichment/shut-in strategy, at Mae Soon site. The relative abundance of predominant taxa found in the oil-bearing sandstone core samples that had been submerged in the reservoir’s produced water (control samples) showed that *Deinococcus-Thermus* (Deinococci) was the most abundant bacterial class in half (three out of six) of the samples tested. As for the other three samples, even though the *Deinococcus-Thermus* (Deinococci) was present, the predominant bacteria differed. *Betaproteobacteria* was predominant in one sample (C1P), while in samples C5P and C6P, *Firmicutes* (Clostridia) and *Acidobacteria* (Solibacteres), were more abundant than others. This is interesting because *Gammaproteobacteria* had been found to be a more-common predominant bacterium in many other studies of oil-reservoir microbial communities than *Deinococcus-Thermus* [31]. Thus, the bacterial communities in the cores submerged in the produced water for a prolonged period of time, being made up largely of *Deinococcus-Thermus* (Deinococci), was different from those related to the oil reservoirs previously reported [32, 33]. The microbial structure of the cores drilled from the oil wells within the reservoir shared some common patterns, yet the core from each oil well preserves its own microbial composition.

At the genus level, sequencing of 16S rRNA gene amplicons from the reservoir samples revealed several potential bacteria for the microbial recovery process including *Thermus*, *Pseudomonas*, *Acinetobacter*, *Bacillus*, and *Clostridium* [34]. The most abundant genus was *Thermus*, which was found 27.3%, 20.0%, 47.3%, 74.5% and 28.5% in samples C1P, C2P, C3P, C4P, and C6P, respectively. This bacterium is generally found under elevated temperature conditions. It can grow at temperatures between 50 to 75°C (with the optimum temperature of 70°C), and is also found in high-temperature petroleum reservoirs (with the temperature range of 50 to 80°C) such as East Paris Basin (France) and Samotlor oil reservoir (Russia) [35, 36]. Many thermophilic bacteria secrete biosurfactants which can support microbial hydrocarbon degradation [37].

Many facultative anaerobic bacteria found in the reservoir samples were bacteria that can potentially support the MEOR process. The *Bacillus* genus was found in sample C1P, C3P, and C6P. This genus occurred in the environment of many oil reservoirs [38]. Some members of this genus are thermotolerant, capable of producing biosurfactants that play an important role in mobilising entrapped oil, and able to survive in extreme conditions. The characteristics and capabilities of this genus are desirable in MEOR processes [39]. Besides these, *Clostridium* was also found, although not in a great abundance. This organism is one of the bacteria that can produce solvents and gases under anaerobic conditions, which would support the efficiency of oil recovery by reducing oil viscosity [2].

There were some changes in the relative abundances of predominant taxa in the core samples after being treated with inorganic nutrient. *Deinococcus-Thermus* (Deinococci) did not seem to be influenced by nutrient enrichment and remained the most abundant class in the reservoir’s community; comprising 41.8%, which was similar to the control group (40.9%). However, when considering the bacterial composition in groups of individual wells, the abundances of this class were significantly different. It was more abundant in C2N, C4N, and C5N than in the controls. This group of bacteria is more commonly present in high temperature environments [40]. Interestingly, *Deinococcus-Thermus* had not been reported to be present in sandstone-based rock; it had only been found in the rock from a coal bed methane reservoir, although in a small proportion [41].

Differences of relative proportions of bacteria as a result of nutrient treatment were observed with *Gammaproteobacteria*, which became the second most abundant group in some nutrient-treated samples. However, because of the high level of variation in the proportions of this group among the samples, the overall changes were not significant. In contrast, *Betaproteobacteria*, *Acidobacteria* (Solibacteres), *Firmicutes* (Clostridia), and *Firmicutes* (Bacilli), were the taxa that experienced a lower abundance than in the control group.

Bacterial community profiles of nutrient-treated sandstone samples determined in this work were clearly different from those of the liquid phase of an offshore reservoir, as reported by Kotlar *et al*. (2011), which were dominated by *Delta/epsilon*-*Proteobacteria* (sulfate-reducing bacteria) and methanogens (mostly *Methanococcus* species) [9]. In our study, *Delta/epsilon*-*Proteobacteria* occurred in low abundance, while methanogens were not found. Although this is an extreme comparison between an onshore and offshore (marine) environment, between sandstone and liquid phase samples, and between the different treatments the samples received; it is clear that bacterial distribution in an environment can be greatly influenced by unique reservoir characteristics, such as NaCl concentration, types of organic compounds present, and reservoir pressure. Even the individual characteristics of the oil wells in the reservoir could also contribute to the specificity of microbial population.

At the genus level, the bacterial genera that were predominant in the nutrient-treated samples were mainly *Thermus*, *Pseudomonas*, and *Acinetobacter*. Again, the overall changes were not significant due to the high level of variation among the samples, but they increased significantly in some samples extracted from some wells. A previous study performed on inorganic nutrient-enriched production fluids resulted in different major microbes, including bacteria in the order *Thermotogales*, methanogens related to *Methanobacterium* and *Methanococcus*, and sulfur-utilising archaea of the genus *Thermococcus* [42]. Nutrient treatment processes may give different results in bacterial community structures and patterns of distribution changes, depending on the original seed culture, type of nutrient used, and conditions and time of incubation. Apart from these, the physical and chemical properties of the oil-bearing strata and the properties of produced water of reservoirs may have significant contributions to the bacterial distribution patterns. To design a nutrient treatment process for a reservoir, which carries its own complexity, all these factors must be taken into account.

In MEOR-related terms, the major bacteria found in the cores after nutrient enrichment were considered having high potentials. *Thermus*, which was the most abundant genus in the treated samples, is known as thermophilic hydrocarbon-degraders [43]. This organism was able to secrete rhamnolipids, a biosurfactant that can directly hydrolyse oil [44]. This genus converted crude oil into lighter hydrocarbons by decreasing aromatics, resins and asphaltenes in crude oil [45]. It is, therefore, one of the promising thermophilic bacteria for MEOR application.

*Pseudomonas*, the second most abundant genus that became one of the predominant groups as a result of nutrient treatment, is capable of producing many kinds of biosurfactant molecules. Biosurfactants can lower interfacial tension of water, thus allowing mobilisation of bound hydrophobic molecules, which, in this case, is the oil trapped in the reservoir rock. The biosurfactants produced by this organism had a high potential for the recovery of crude oil [46]. Besides, *Pseudomonas* is known as a hydrocarbon-degrading bacterium, and it is effective in stabilising oil-in-water emulsion that can improve the ability of oil recovery [47]. In addition, *Pseudomonas* was applied, together with other microbes, to enhance oil recovery rate in the Bragadiru oil field, which caused the increase in oil production from 1.5 to 7.4 bbl/d per well [48]. The existence of *Pseudomonas* in onshore reservoirs had also been observed in other studies [31, 42]. *Pseudomonas* was found in the produced water of Gudao oil reservoir, China [49] and of Daqing oil field, China [33]. Fluid samples collected from producing wells of an offshore petroleum reservoir also contained a high proportion of this organism [32]. The fact that *Pseudomonas*, which is typically aerobic, was found in oil reservoirs was interesting. This genus could grow in an anaerobic condition, as reported by Kerschen *et al*. (2001), since it could use nitrate as a terminal electron acceptor for both assimilation and anaerobic respiration [50].

*Acinetobacter*, another genus present in a large proportion as a result of nutrient treatment, is another potential bacterium for a MEOR process. Several species of *Acinetobacter* produce substances involved in emulsification, interfacial tension reduction, and oil viscosity reduction [51]. These abilities are potentially useful for enhanced oil recovery. Metabolites from this genus have been used to increase displacement efficiency [34]. Previous studies have shown that this organism was found in produced water of Daqing oil field and in crude-oil-contaminated soil of Zhongyuan oil field, China [33, 52].

Several other bacterial genera such as *Clostridium*, *Klebsiella*, and *Bacillus*, known as potential producers of some metabolites that can enhance oil recovery, were also detected, although in low proportions. *Clostridium* and *Klebsiella* have been reported to increase the oil recovery process by reduction of oil viscosity [2]. *Bacillus* is among the organisms with high potentials to produce biosurfactants and enzymes [53, 54]. Members of this genus could lower the interfacial tension of water, and thus could allow water to mobilise residual oil trapped in a reservoirs’ oil-bearing zone. The spore-forming capability of *Bacillus* is also favourable for its survival in harsh conditions of different reservoirs [39]. This genus, however, was found in relatively low proportion in our study, even lower after the nutrient treatment. The treatment using inorganic nutrients that could cause some bacterial genera, such as *Thermus*, *Pseudomonas*, and *Acinetobacter*, to prevail, obviously could not promote the growth of *Bacillus*. It could be possible that the organism remained in the dormant stage under the nutrient-treated conditions given in this study. Conditions that can elevate this genus to a higher relative abundance remain to be further investigated.

Some bacterial genera in the reservoir’s communities can be influenced by reservoir parameters, such as temperature and depth of the wells, grain density and permeability of the cores, and chemical compositions of the cores. Inorganic nutrient composition could also alter bacterial population, although the patterns of change were not consistent in individual wells. Since predominant bacterial genera could greatly influence biosurfactants, gases, acids, solvents, and biopolymers production, it is important to consider the connection between physical/chemical factors of the reservoir and the microbial structure, in order to evaluate the suitability of the reservoir for MEOR application and to appropriately design a MEOR strategy.

## Conclusion

This study demonstrated how the bacterial structure in oil-bearing sandstone cores extracted from Mae Soon reservoir, Fang Basin, Thailand, and how the additional inorganic nutrients, together with the physical and chemical characteristics of the reservoir, were related. Bacterial community analysis of oil-bearing sandstone cores submerged in the produced water under high temperature conditions revealed *Deinococcus-Thermus* (Deinococci) and *Betaproteobacteria* as the predominant groups. The bacterial communities in samples extracted from some wells of the reservoir were altered after the inorganic nutrient treatment, resulting in a different bacterial population, most obviously in *Betaproteobacteria* giving dominance to *Gammaproteobacteria*, while *Deinococcus-Thermus* remained the most abundant group. At the genus level, *Thermus*, *Pseudomonas*, and *Acinetobacter* were the most abundant. Members of these genera are capable of producing hydrocarbon-degrading enzymes, surfactants, and other MEOR-related substances, which are desirable for MEOR application. The microbial communities were supported by the reservoir’s physical/chemical characteristics.

This experiment was set to address whether the inorganic nutrient treatment would have an effect on the reservoir’s bacterial community as a whole, or on the communities in the individual wells, so that MEOR could be designed according to the finding. Our results seemed to point that, for this reservoir, such nutrient treatment may be potential, but the application of MEOR processes might be considered in individual wells rather than one process for the whole reservoir’s system. However, whether or not the field application based on this laboratory finding would be effective, *in situ* investigation must be performed. Nevertheless, the approach of this study in employing the information of a reservoir’s bacterial communities, fortified with inorganic nutrients as necessary, together with the reservoir’s physical and chemical properties, can be considered for a preliminary evaluation of the possibility of conducting or for designing a MEOR process.

## Supporting information

S1 File The statistical analysis supporting the results are shown in Fig 2A and 2B. The rarefied OTUs (16,828) were obtained by subsampling method and statistical calculation was performed using the non-parametric Mann-Whitney U test.
(XLSX)

S1 Table The statistical testing of the difference between unweighted and weighted Unifrac data. The rarefied 16,828 OTUs (Fig 2C) were used for statistical analysis, which was performed by PERMANOVA to compare the difference between two treatments.
(XLSX)

S2 Table Spearman’s correlation analysis. The statistical results show significant correlations between physicochemical factors and bacterial genera in the reservoir communities.
(XLSX)
